# The Queen and I: Neural Correlates of Altered Self-Related Cognitions in Major Depressive Episode

**DOI:** 10.1371/journal.pone.0078844

**Published:** 2013-10-30

**Authors:** May Sarsam, Laura M. Parkes, Neil Roberts, Graeme S. Reid, Peter Kinderman

**Affiliations:** 1 Institute of Psychology, Health and Society, University of Liverpool, Liverpool, United Kingdom; 2 Centre for Imaging Sciences, Institute of Population Health, University of Manchester, Manchester, United Kingdom; 3 Clinical Research Imaging Centre (CRIC), University of Edinburgh, Edinburgh, Scotland, United Kingdom; 4 Division of Health Research, Lancaster University, Lancaster, United Kingdom; The University of Melbourne, Australia

## Abstract

**Background:**

Pervasive negative thoughts about the self are central to the experience of depression. Brain imaging studies in the general population have localised self-related cognitive processing to areas of the medial pre-frontal cortex.

**Aims:**

To use fMRI to compare the neural correlates of self-referential processing in depressed and non-depressed participants.

**Method:**

Cross-sectional comparison of regional activation using Blood Oxygen Level Dependent (BOLD) fMRI in 13 non-medicated participants with major depressive episode and 14 comparison participants, whilst carrying out a self-referential cognitive task.

**Results:**

Both groups showed significant activation of the dorsomedial pre-frontal cortex and posterior cingulate cortex in the ‘self-referent’ condition. The depressed group showed significantly greater activation in the medial superior frontal cortex during the self-referent task. No difference was observed between groups in the ‘other-referent’ condition.

**Conclusions:**

Major depressive episode is associated with specific neurofunctional changes related to self-referential processing.

## Introduction

Both psychological therapy and medication are commonly used in the treatment of major depressive episodes, and have been found to be roughly equally effective [1]. Despite this, their modes of action are currently seen as quite distinct [2]. Functional brain imaging allows the neural correlates of distinct cognitive processes to be revealed through regional brain activity by asking participants to carry out specifically designed tasks during scanning. Such methods are increasingly used to investigate the neurofunctional markers associated with mental health problems and recovery [3], [4]. Functional brain imaging also allows researchers scope to investigate the neural correlates of the specific cognitive and emotional processes known to be important in psychological models of mental disorder. Incorporating psychological models into the interpretation of functional brain activity has several important advantages for both research and treatment, and is an important ‘next step’ in our understanding of the neural correlates of mental health.

Psychologically, major depressive episodes are associated with widespread and pervasive information-processing and cognitive biases in the functioning of self-related attentional, memory and attribution systems [5], [6]. Neuroimaging research conducted with the general population has consistently reported self-related information processing (self referential processing) to occur in specific areas in the pre-frontal cortex, known collectively as Cortical Midline Structures (CMS) [7], [8]. This includes the orbito-medial-pre-frontal cortex (OMPFC), the dorso-medial prefrontal cortex (DMPFC), the anterior cingulate (AC) and posterior cingulate (PC).

In several treatment outcome studies, the cortical areas showing functional changes on remission of depressive episode following both pharmacological and psychological treatment have included the posterior cingulate, anterior cingulate gyrus, orbitofrontal and medial prefrontal areas or the dorsolateral, ventrolateral, and medial aspects of the prefrontal cortex [1], [9], [10]. CMS changes have also been reported in studies comparing self-referential and non-self referential processing in depressed people, including functional changes in the dorsolateral prefrontal cortex and dorsal part of the medial frontal gyrus [11], [12]. Self-referential processing in medicated depressed people have been found to be associated with activation in the medial prefrontal cortex and rostral anterior cingulate cortex in response to either negative stimuli only [13] or both positive and negative stimuli [11].

The present study therefore aimed to extend the evidence-base concerning the neural characteristics of self versus other-referential processing in depression, comparing the pattern of processing in the general population and in unmedicated people experiencing major depressive episodes. Given the increased attentional bias to negative self-related information in depression, it was hypothesised that participants currently experiencing a depressive episode would show increased regional brain activity during a self-referential task compared to an other-referential task and in comparison with a never-depressed control group. Given the potentially confounding effects of antidepressant medication on CMS functioning [1], [9], [10], [13], the current study recruited depressed participants who were free from antidepressant medication.

## Method

### Design

Functional-MRI, using the Blood Oxygen Level Dependent (BOLD) technique, allows regional brain activity during a target cognitive task to be compared with a baseline condition. Differential BOLD response between the two conditions is localised throughout the brain, allowing the observation of specific anatomical areas of neuronal activity associated with the cognitive task being investigated. Using a well established study design [14], we contrasted the BOLD response in medication-free participants with major depressive episode and never-depressed controls, while making judgements on whether adjectives described themselves (self-referent condition) or the British Queen (other-referent condition). Our design improved on previous studies by introducing measures to minimise habituation effects and observe neural correlates of task adherence. The other-referent target, the British Queen, was chosen as a universally well-known figure about whom participants would be able to make evaluative judgements with relative ease.

### Setting

The study was conducted in a specialist neuroimaging laboratory in the University of Liverpool, UK, with participants recruited from local mental health services and the local community.

### Ethics Statement

This research was approved by Liverpool NHS PCT Local Research Ethics Committee (ref 05/Q1505/10), and was conducted according to the principles expressed in the Declaration of Helsinki, including fully informed, written, consent. All clinical participants were interviewed by a mental health professional involved in their care, who ensured that they had capacity to understand the ethical issues, and nurses experienced in the ethical procedures involved in neuroimaging performed a similar assessment in the case of control participants.

### Participants

Following ethical approval, participants with a current diagnosis of major depressive episode (Depressed group) were recruited through advertisements in GP surgeries within Central and South Liverpool Primary Care Trusts. Participants with no history of mental health difficulties were recruited via advertisements in the local media (Control group). Age of the depressed group was (mean ± SD) 32.7±7.6 years. Age of the control group was 26.4±9.5 years. There was no significant difference between the age of the groups (p = 0.1, 2-sample t-test). Mean number of years of education was 15 years in both groups ± 2.6 in the depressed group and ± 3.0 in the control group. Inclusion criteria were a Beck Depression Inventory II (BDI-II) [15] score of 15 or above in the depressed group, or 7 or below in the control group. Participants in both groups were required to be between ages 18-65, right handed, score 6 or less reading errors on the National Adult Reading Test (NART) [16], and speak English as their first language. Specific exclusion criteria for both groups included: epilepsy, major medical or neurological disorders, cerebro-vascular abnormalities, brain injury, history of alcohol or substance misuse, history of premature birth below 32 weeks, and use of non-inhaled steroids or benzodiazepines. The depressed group were required to be free from anti-depressant medication in the current episode of depressed mood and for the last 6 months and free from participation in any psychological therapies for the same length of time. They were also required to have no co-morbid mental health difficulties. All exclusion criteria were checked by a telephone screening interview prior to formally recruiting participants to the study. Participants were paid £25 for participation.

Participants received a physical examination from MRI nurses to confirm that there were no contraindications for brain imaging. This included pulse and blood pressure check, Romberg’s test of cerebellar function, Edinburgh Handedness Inventory, and weight and height check. Participants then completed a Structured Clinical Interview for DSM-IV (SCID) [17] to assess for Axis I disorders, the BDI-II, the Rosenberg Self-Esteem Inventory [18], and the NART [16]. None of the participants in the control group had a history of mental health difficulties. All members of the depressed group currently met DSM-IV criteria for ‘major depressive episode’. Two participants from each group did not complete the scanner task and were therefore excluded from the analysis. The final sample consisted of 13 depressed group participants (10 female) and 14 control group participants (8 female).

### Image Acquisition

All scanning was performed on a 3-Tesla Siemens whole-body Trio system. An 8-channel phased array head coil was used to receive the MRI signal. For the fMRI scanning, a standard single-shot echo-planar imaging (EPI) sequence was used with the following acquisition parameters: repetition time 2s, echo time 35ms, matrix size 64 x 64 with a field of view of 224 mm to give in-plane pixel resolution of 3.5 mm. Twenty eight slices with a slice thickness of 3.5 mm and a 10% gap allowed full coverage of the cerebral cortex. Prospective motion correction [19] was used to reduce movement-related signal changes. An MPRAGE [20] sequence with a 1 mm isotropic resolution was used for the structural scanning.

### Self-Referential Processing Task

Functional brain imaging methodologies for investigating self-referential processing are well established [7], [8], [14]. While early designs failed to adequately control for auditory, attentional and motor demands, subsequent studies have progressively standardised the design parameters between conditions, also controlling for ‘depth’ of information processing which may confound a comparison between making evaluative judgements about the self (such as describing one’s own attributes) versus making a concrete comparator judgement (such as whether a concrete statement is true or false) [21].

The methodology used by Kelley and colleagues [14] allowed a self-referent condition to be compared with an other-referent condition where the ‘other’ was a public figure familiar enough to the majority of the population to enable participants to make character judgements based on their experience. The design controlled for auditory and attentional demands. As both conditions required participants to make evaluative judgements (either about themselves or the public figure), the semantic demands of the two conditions were also considered to be well-matched. Kelley and colleagues’ design was therefore adopted as a template for the current fMRI task, using the British Queen as the familiar ‘other’. Use of the British Queen as the object of our other-referent condition provided continuity of stimulus design with several comparable studies [13], [22], [23]. Participants were required to indicate with a yes-no response button if personality descriptor words described themselves (‘self’ condition), or described the British Queen (‘queen’ condition). The fMRI tasks were considered equally accessible to both depressed and non-depressed groups.

Stimulus words were chosen from within Anderson’s list of 555 personality descriptors [24]. Words were chosen for having highly positive, negative or neutral valence scores, high ‘meaningfulness’ scores, and a frequency in the English language greater than 1 per 100,000 words. The number of syllables in each set of words was balanced, leaving 78 stimulus words – 26 positive, 26 negative and 26 neutral. All stimulus words were used in each of the two main study conditions (‘self’, and ‘queen’), as well as in a third, control condition described below, making a total of 234 trials.

**Figure 1:**
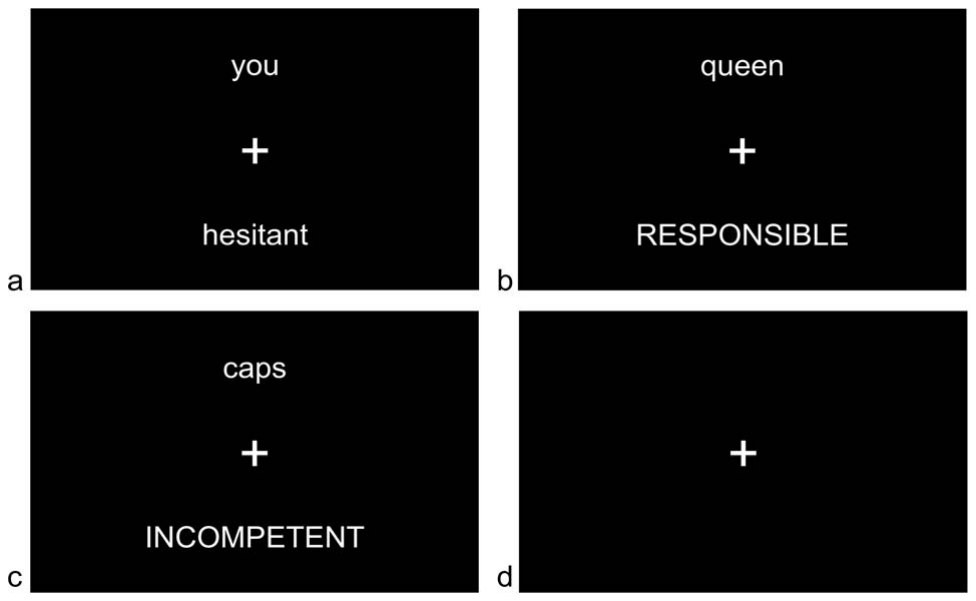
fMRI stimulus presentation. a =  self condition, neutral stimulus word; b = other condition, positive stimulus word; c = case condition, negative stimulus word; d = Rest Interval.

The visual stimuli were controlled via Presentation software (Neurobehavioural Systems Inc.) [25] and consisted of a fixation cross with the stimulus word presented underneath the cross and an instruction word for participants (either SELF, QUEEN or CAPS) presented above (see *figure 1* *)*. Words were presented for 3 seconds separated by a fixation cross presented for a duration of 3.5, 4, or 4.5 seconds (mean 4 seconds). This jittering allowed for optimal sampling of the BOLD response. A total of 234 words were presented in a randomised order over 3 separate runs each of which lasted 9 minutes 10 seconds. The event-related design of the study meant that conditions were also selected in randomised order. While this design meant that condition-related habituation effects were minimised, asking participants to ‘switch’ between cognitive tasks every few seconds may be demanding. A third condition was therefore added to allow a check of task adherence. In this third condition, participants indicated whether the stimulus word was printed in upper case letters (‘case’ condition), providing a task of distinctly different complexity, salience, and emotiveness compared to the two evaluative experimental tasks. This task was expected to show a distinctly different pattern of functional activity, which would be observable in statistically significant BOLD signal change.

The stimuli were back-projected onto a screen at the rear of the scanner bore and were viewed via a head-coil mirror. Participants were first given a two minute practice run (using target words not appearing in the full stimulus list) inside the scanner. The three functional runs and an 8 minute structural image were then collected.

### Behavioural Data Analysis

Mean positive responses (percentage of responses answered ‘yes’) and response times were calculated for each condition for each participant. Mean positive responses and response times that were more than 3 x SD from the global mean (control and depressed participants taken together) were discarded (0.5% of data). A ‘positivity measure’ was defined as: % response ‘yes’ to positive valence words - % response ‘yes’ to negative valence words. Comparisons between groups were made using two-sample t-tests for all 9 conditions. In addition, difference in response time for (self – queen) and (self – case) and the ‘positivity measure’ for self and queen were compared between groups to test for specific differences in self-referential processing. For the response times, a 3-way ANOVA with group (control or depressed), category (queen, self or case) and valence (positive, negative or neutral) was performed in Matlab (The Mathworks, Inc, Massachusetts, USA). Linear regression between (self – queen) response time and the ‘positivity measure’ were made with the BDI II and Rosenberg scores to determine if these measures related to depressive symptoms.

### fMRI Data Processing and Analysis

All images were analysed using BrainVoyager software (Brain Innovation, Maastricht). Pre-processing comprised motion correction, slice time correction, spatial smoothing (6 mm FWHM 3D Gaussian filter) and temporal filtering (linear trend removal and 0.02 Hz high pass filtering). The fMRI images were co-registered onto the structural images and transformed into Talairach space.

A general linear model was constructed for each fMRI run of each subject with 9 regressors: 3 valences of word (positive, negative and neutral) x 3 categories of presentation (self, queen and case). A random effects analysis was performed on both the depressed group of 13 subjects and the control group of 14 subjects separately. The contrast (self – queen) was considered collapsed across all 3 word valences. Results pertaining to individual valences were not considered as there would be too few trials of each condition to provide reliable results. Results were thresholded at p<0.005 uncorrected with a cluster threshold set to 8 voxels (voxel here refers to the original size in the fMRI data, one voxel  =  47 mm^3^). This cluster-size method to correct for multiple comparisons avoids the potential over-correction of more conservative tests such as Bonferroni. A fairly lenient statistical threshold was chosen to avoid type II errors at the risk of type I errors. The large cluster size (approximately 400 mm^3^) effectively guards against type I errors, as any ‘activation’ arising by chance would be expected to be scattered through the brain. This is perhaps at the expense of missing very small regions of true activation. This choice of cluster threshold is supported by the simulation work of Lieberman and Cunningham [26], who suggest that a combined intensity and cluster size thresholds such as P<0.005 with a 10 voxel extent produce a desirable balance between Types I and II error rates. Talairach coordinates and the mean BOLD signal amplitude in the significantly active regions within grey matter were recorded.

### Depressed Group versus Control Group Comparison

In a separate analysis, the difference in activation (self - queen) between the two groups was compared in a random effects analysis. A two-stage procedure was used for this where a first-level fixed effect analysis was used to determine the effect size (self-queen) in each subject. A second-level random effects analysis then compared this effect size between the two groups across the brain. Results were again thresholded at p<0.005 uncorrected with a cluster threshold of 8 voxels. Talairach coordinates were recorded and the BOLD signal amplitude of each of the groups in the active grey matter regions and the significance of any difference was recorded. Linear regression between the BOLD amplitude and the BDI and Rosenberg scores were performed. ‘Self-case’ and ‘queen-case’ contrasts were also analysed, using the same procedure as for ‘self-queen’ above.

## Results

### Participants

Depressed participants scored a mean of 29.1±12.9 on the BDI-II (a score of 20 or above on the BDI-II is indicative of at least moderate depression) and a mean of 10.9±4.6 on the Rosenberg self-esteem inventory (a score of 10 or below on the Rosenberg is indicative of clinically significant low self-esteem). The control group scored a mean of 3.3±2.5 on the BDI-II and 24.8±4.0 on the Rosenberg. Both the BDI-II and Rosenberg scores differed significantly between the two groups at p<0.001 (t(26)  =  7.34 for BDI-II scores, t(26)  =  8.47 for Rosenberg scores.

### Behavioural responses

In order to examine possible demand characteristics, the behavioural responses of participants to the stimulus questions were analysed. Overall participants recorded a response (i.e either a ‘yes’ or a ‘no’ response) on 98.7% of occasions. There was no significant difference between the number of depressed and control participants’ responses. Both groups responded swiftly to the stimuli materials, with the control group responding significantly faster (1.4±0.2 seconds) overall than the depressed group (1.8±0.2 seconds) (t (26)  =  3.23, p<.0005). A summary of response times and endorsement rates, along with results of 2-sample t-tests between the control and depressed groups are presented in Tables 1 and 2.

**Table 1:** Response times (ms).

Category	Valence	Control	Depressed	Cont vs Clin
		Mean ± SD (ms)	Mean ± SD (ms)	p-value
Self	Positive	1317±276	1779±205	<0.0001
	Neutral	1565±137	1921±192	0.03
	Negative	1344±251	1859±166	0.007
Queen	Positive	1624±281	1824±195	0.0006
	Neutral	1720±216	1966±238	<0.0001
	Negative	1520±301	1783±178	0.007
Case	Positive	1260±208	1583±350	<0.0001
	Neutral	1248±189	1616±295	0.007
	Negative	1245±265	1573±327	0.006
Self - Queen	All	−213±108	−5±115	<0.0001
Self - Case	All	158±128	310±166	0.01
Self	Neg – Pos	62±93	80±167	0.7

**Table 2:** Percentage of responses answered ‘yes’.

Category	Valence	Control	Depressed	Cont vs Clin
		Mean ± SD (ms)	Mean ± SD (ms)	p-value
Self	Positive	93±12	77±16	0.01
	Neutral	37±17	56±12	0.004
	Negative	6±7	41±22	0.001
Queen	Positive	63±29	76±22	0.2
	Neutral	29±12	33±10	0.4
	Negative	21±24	17±17	0.6
Case	Positive	49±3	51±8	0.4
	Neutral	50±3	52±5	0.2
	Negative	52±6	53±4	0.8
Self	Pos – Neg	87±16	36±36	0.0004
Queen	Pos - Neg	34±30	43±24	0.4

A 3-way ANOVA of response times revealed main effects of all factors (group F = 131 p<0.0001; category F = 40 p<0.0001; valence F = 6.5 p = 0.002). There was also a significant interaction between group and category (F = 5.3, p = 0.005) but not between group and valence. The results show that the depressed group were significantly slower than controls in their response to all stimuli (Table 1). In addition, the depressed group were specifically slower than the control group for the ‘self’ category, relative to the other categories. There was no evidence of negative bias in the response times.

Table 2 shows the relative endorsement rates of the different categories. The depressed group gave significantly different responses compared to the control group for the ‘self’ category only. They attributed fewer of the positive traits to themselves and more of the neutral and negative traits. The ‘positivity measure’ (Pos – Neg) was significantly lower for the depressed group for the ‘self’ condition only.

Significant correlations were found between the (self – queen) response times and the BDI II scores (r^2^ = 0.38, p = 0.002) and Rosenberg scores (r^2^ = 0.36, p = 0.002) across the cohort. However, these were not significant within the depressed group alone (BDI II: r^2^ = 0.01, p = 0.8; Rosenberg: r^2^ = 0.02, p = 0.6). Similarly, the ‘positivity measure’ for the self condition showed significant correlations with BDI II scores (r^2^ = 0.65, p<0.0001) and Rosenberg scores (r^2^ = 0.56, p = 0.0001), but again not within the depressed group alone (BDI II: r^2^ = 0.25, p = 0.1; Rosenberg: r^2^ = 0.31, p = 0.1). The strength of these correlations however, suggests that the ‘positivity measure’ may be related to depressive symptoms and the lack of significance may be due to the small sample size.

### BOLD response

**Figure 2:**
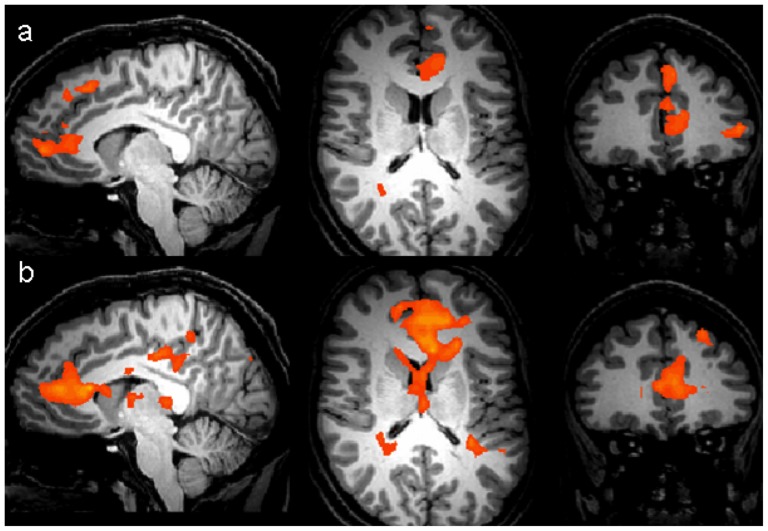
Sagittal, axial and coronal views of areas of statistically significant BOLD activation (p = 0.005 uncorrected, cluster threshold 8 voxels) for the condition ‘self – queen’ in (a) the depressed group (n = 13) and (b) the control group (n = 14). Areas in red represent regions of increased BOLD activity in the ‘self’ as opposed to ‘queen’ condition.

Figure 2 shows the sagittal, axial and coronal views of the BOLD activation for the contrast ‘self – queen’ across all 13 depressed participants (a) and 14 control participants (b). The active regions (in red, shown at a statistical threshold of p = 0.005 uncorrected with a cluster threshold of 8 voxels) represent regions where the ‘self’ condition resulted in significantly greater BOLD signal change than the ‘queen’ condition. The anatomical areas showing statistically significant activation are listed in Tables 3 and 4.

**Table 3:** Regions exhibiting significant ‘self – queen’ activity in the depressed group†.

Region	Talairach coordinates x y z	Volume mm^3^	BOLD signal (se)*	T-value of region	p value of region
Medial superior frontal gyrus	−1 31 40	2366	1.45 (0.12)	11.8	<.000001
Medial frontal cortex & anterior cingulated	−4 36 11	3870	1.25 (0.11)	11.6	<.000001
Left inferior frontal gyrus	−42 29 7	1064	0.74 (0.11)	6.9	<.000001

† Significant at the p = 0.005, uncorrected significance level with a cluster threshold of 8 voxels.

* The BOLD signal amplitude for the (self – queen) condition is given, taken from the beta weights of the general linear model fit. ‘se’ is the standard error over all participants.

**Table 4:** Regions exhibiting significant ‘self – queen’ activity in the control group†.

Region	Talairach coordinates x y z	Volume mm^3^	BOLD signal (se)*	T-value of region	p value of region
Medial frontal cortex & anterior cingulated	−6 33 14	5369	1.1 (0.1)	10.4	<.000001
Cingulate gyrus (central and posterior)	21 −15 24	3037	0.8 (0.1)	7.7	<.000001
	−7 −33 35	2506	00.78 (0.1)	77.5	<.000001
	−16 0 27	552	0.7 (0.1)	76.7	<.000001
Right superior temporal gyrus	50 −31 4	2723	0.67 (0.1)	6.4	<.000001
Caudate body	0 −8 12	711	0.81 (0.1)	7.7	<.000001
Bilateral thalamus	7 −32 3	641	0.58 (0.1)	5.6	<.000001
Left parahippocampal gyrus	−42 −27 −8	639	0.62 (0.1)	5.9	<.000001
Precuneus	−2 −44 47	455	0.53 (0.1)	5.1	<.000001
Left superior frontal gyrus	−21 31 46	519	0.75 (0.1)	7.2	<.000001
Left cerebellum	−30 −49 −15	627	0.4 (0.1)	3.9	.0001
	−38 −65 −18	942	0.58 (0.1)	5.8	<.000001
Right brainstem	6 −26 −18	551	0.44 (0.1)	4.2	0.0002

† Significant at the p = 0.005, uncorrected significance level with a cluster threshold of 8 voxels.

* The BOLD signal amplitude for the (self – queen) condition is given, taken from the beta weights of the general linear model fit. ‘se’ is the standard error over all participants.

As can be seen from Figure 2 and Tables 3 and 4, both depressed and control groups showed extensive differential ‘self – queen’ activation in the medial frontal cortex.

### Depressed Group versus Control Group Comparison

**Figure 3:**
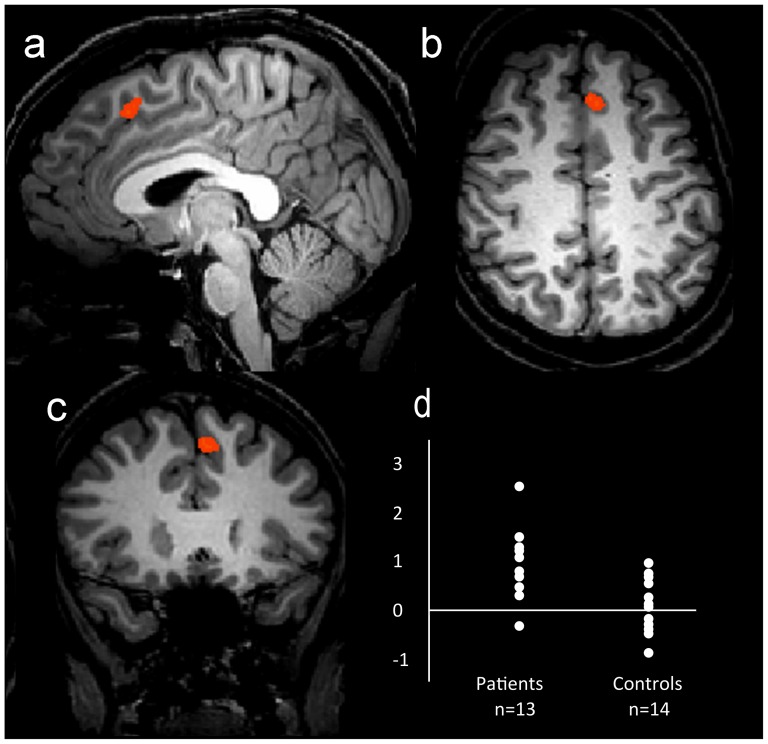
Sagittal (a), axial (b) and coronal (c) views of areas with statistically significant BOLD activation (p = 0.005 uncorrected, cluster threshold 8 voxels) for the comparison of the ‘self – queen’ contrast between the depressed and control groups. (d) represents the statistical distribution of the BOLD signal change in the highlighted area. The areas in red represent areas where the depressed participants showed significantly greater BOLD signal change for the contrast ‘self – queen’ than the control group.

The pattern of activation revealed by the ‘self – queen’ contrast was compared between the two participant groups. Figure 3 shows the sagittal (a), axial (b) and coronal (c) view of the regions showing different BOLD activation between the depressed and control groups for the contrast ‘self-queen’. The active regions (in red) represent regions where the depressed participants showed significantly greater BOLD signal change for the contrast ‘self – queen’ than the control group, in the direction of the ‘self’ condition. Despite the apparently more widespread activation observed in the control group in Figure 2, the random effects analysis showed no areas of significantly greater BOLD signal change in the control group. The activated regions refer to a statistical threshold of p = 0.005 uncorrected with a cluster threshold of 8 voxels. As shown in Figure 3, one specific region, the medial superior frontal cortex (−1,21,48), showed significantly different BOLD signal change relative to baseline between the two groups (see Table 5). Significant correlation was found between the (self – queen) BOLD amplitude in this region and the BDI II scores (r^2^ = 0.26, p = 0.01) and Rosenberg scores (r^2^ = 0.26, p = 0.01) across the cohort. However, these were not significant within the depressed group alone (BDI II: r^2^ = 0.01, p = 0.9; Rosenberg: r^2^ = 0.01, p = 0.7).

**Table 5:** Regions exhibiting significantly increased ‘self – queen’ activity for the depressed group compared to the control group†.

			BOLD signal (se)*	BOLD signal (se)*		
Region	Talairach coordinates x y z	Volume mm^3^	Clinicals (n = 13)	Controls (n = 14)	T value of region	p value of region
Medial superior frontal cortex	−1 21 48	528	0.72 (0.2)	−0.06 (0.12)	3.6	0.001

† Significant at the p = 0.005, uncorrected significance level with a cluster threshold of 8 voxels.

* The BOLD signal amplitude for the (self – queen) condition is given, taken from the beta weights of the general linear model fit. ‘se’ is the standard error over all participants.

### Further Analysis

Task adherence analysis was conducted by comparing BOLD signal change in the ‘self’ and ‘queen’ tasks with the simple case-judgement task. Both self-case and queen-case comparisons revealed patterns of differential activation that were not statistically different between the two participant groups, providing confirmation that both the depressed and control participants adhered to the demands of each randomly presented task. The lack of activation for self-case and queen-case comparisons gives confidence that the statistical threshold is appropriate to avoid type I errors.

## Discussion

This investigation compared BOLD signal change in depressed and non-depressed participants while performing a well-controlled self-referential task. The ‘experimental’ task – the self-referent decision “does this word describe me?” – has good face validity as a stimulus for the investigation of altered self-related cognitions that are implicated in psychological models of depression [5], [6], [15]. This task was contrasted with a well-matched and equivalent (but non self-referent) task – “does this word describe the British Queen?”, and a non-evaluative control task – “is this word written in upper-case letters?” The jittered, event-related presentation of the stimuli meant that timing-related and condition-related habituation effects were minimised. Behavioural data confirmed a high response rate to all stimulus tasks across both groups. The investigation also improved on previous designs by only recruiting non-medicated and non-psychotherapy-receiving depressed participants in order to eliminate the potential confounding effects on cortical functioning. While the sample size is therefore modest, the results have yielded some important clarifications on previous research findings.

Across the two participant groups, the contrast of the ‘self’ versus ‘queen’ tasks revealed activation associated with self-referential processing in the medial superior frontal gyrus, medial frontal cortex and anterior cingulate. Thus far, therefore, the findings of the present investigation are largely consistent with previous findings regarding the nature of self-referential processing in depressed [11], [13], [27] and non-clinical populations [7], [8], [14].

As predicted, there was an increased level of activation in the CMS during self-referent tasks in the depressed group, when contrasting BOLD signal change associated with self-referent processing between the participant groups. When depressed participants made evaluative decisions about themselves, there was significantly greater activation of the medial superior frontal cortex than in the control participants. These findings support and may help to explain several other research studies that have reported higher activation (e.g. blood-glucose metabolism) in specific areas of the orbito, superior and medial prefrontal cortex, and in the anterior cingulate cortex associated with depression, and showed more normalised brain activation after remission following treatment with either medication or psychological therapy [10],[11],[12],[27],[28]. There is a possibility that the increased BOLD response in the medial superior frontal cortex in the depressed group is related to increased attention to the task, as indicated by the longer reaction times. Separation of attentional processing from self-referential processing is difficult in such tasks. However, the contrast ‘self-case’, where reaction time differences were still present, did not reveal any differences in BOLD response between the two participant groups, suggesting that it is specifically self-referential processing that is responsible for the difference in the ‘self-queen’ contrast. In addition, differences in attentional demand might have been expected to activate a wider functional network [29].

Behavioural data in the present investigation showed a distinctly different endorsement profile between the two participant groups. Depressed participants endorsed significantly fewer positive words and significantly more neutral and negative words in the ‘self’ condition as compared with control participants, whereas no significant differences were found in endorsement rates for the ‘other’ or ‘case’ conditions. The ‘positivity measure’ also showed significant correlations between both BDI-II scores and Rosenberg scores. This provides important contextualising information for the BOLD results discussed above. Psychological models predict that depressed individuals will display distinct information-processing and attentional biases in self-related cognition [5], [6]. The results of the present study show a ‘hand in hand’ pattern of behavioural data and corresponding neurofunctional markers, supporting these models and bringing a theoretical framework to the some of the frequently reported differences in CMS activity in depressed populations.

Given that participants in the present study were free from antidepressant medication, it is of interest that there were no significant differences in left dorsolateral cortex activation in response to self-referential processing tasks. This contrasts with neurofunctional changes in response to self-referential processing reported in association with the use of antidepressant medication [27] and is more consistent with reported self-referential functioning changes following Cognitive Behavioural Therapy for depression [12].

Recent models of mental disorder [30], [31] emphasise the necessary integration of psychological, social and biological perspectives. The present study found that major depressive episode was associated with differences in information processing about the self at the neurophysiological level, alongside clear biases towards negative self-related endorsements in the behavioural data. No evidence of differences in regional brain activity was observed in depressed participants’ engagement in decision-making tasks regarding a familiar other, or in a more general cognitive task. The finding offers evidence that some of the observed functional differences in the medial pre-frontal cortex in depression, which normalise in response to both anti-depressant and psychological therapies, may be correlated directly with differences in cognition observed during episodes of depression. This has the potential to offer service users an integrated model of depression which parallels and contextualises both biological and psychological ways of understanding the disorder [30]. 

The lack of observed differences in regional brain activity between the two groups when engaged in non self-related tasks (the ‘Queen’ and ‘case’ conditions) is also interesting from research design and interpretation perspectives. The findings indicate a clear neurofunctional correlate of altered cognitive processing about the self, but are suggestive of a high level of specificity in this altered functioning. In the present study, there was a specific difference in regional brain activity; which fits with psychological models of depression [5], [6] - that people who are depressed process self-related information differently. Both past and future brain imaging research into depression may be more fully understood in the context of this finding. The psychological salience of the task participants are asked to engage in during brain imaging investigations is likely to significantly impact on observed results, and as such, should also be taken into consideration when interpreting content-specific differences in regional brain activity in depression. Further study may determine whether this observation is generalisable to other categories of mental health problems.

### Limitations and further research

This study reported cross-sectional data on a group of people currently suffering from major depressive episode and a group of healthy control participants, and as such provides a ‘snapshot’ of the effects of depression on self-referential processing. Whilst there already is some evidence of specific neurofunctional changes following use of antidepressant medication [27] and CBT [12] that are helpful in generating causal hypotheses, it may be useful to conduct fine-grained prospective research to examine whether changes in self-referential processing occur prior to, concurrently with, or subsequent to changes in mood states. Prospective studies of individuals “at risk” of developing a first or subsequent episode of depression have begun to emerge, for example a recent study indicating a positive correlation between the activation of the dorsal medial prefrontal cortex during self-referential processing and a measure of liability for anxious and depressive disorders among young healthy participants [32]. Further research in this area is needed to determine whether self-referential processing differences may constitute a vulnerability factor or mediating variable that may help explain the onset, topography, remission and recurrence of depression. If self-referent processing is demonstrated to have a causal or mediating role in depression then such findings may contribute to the development of more precisely targeted clinical interventions.
